# Supplementary material for: An Open-Source Pipeline for Processing Direct Infusion Mass Spectrometry Data of the Human Plasma Metabolome
Source: Metabolites. 2022 Aug 21;12(8):768. doi: 10.3390/metabo12080768 (PMC9415960; doi:10.3390/metabo12080768)
Supplement: Supplementary file 1 [file Figure S1.pdf › Figure S1.pdf]

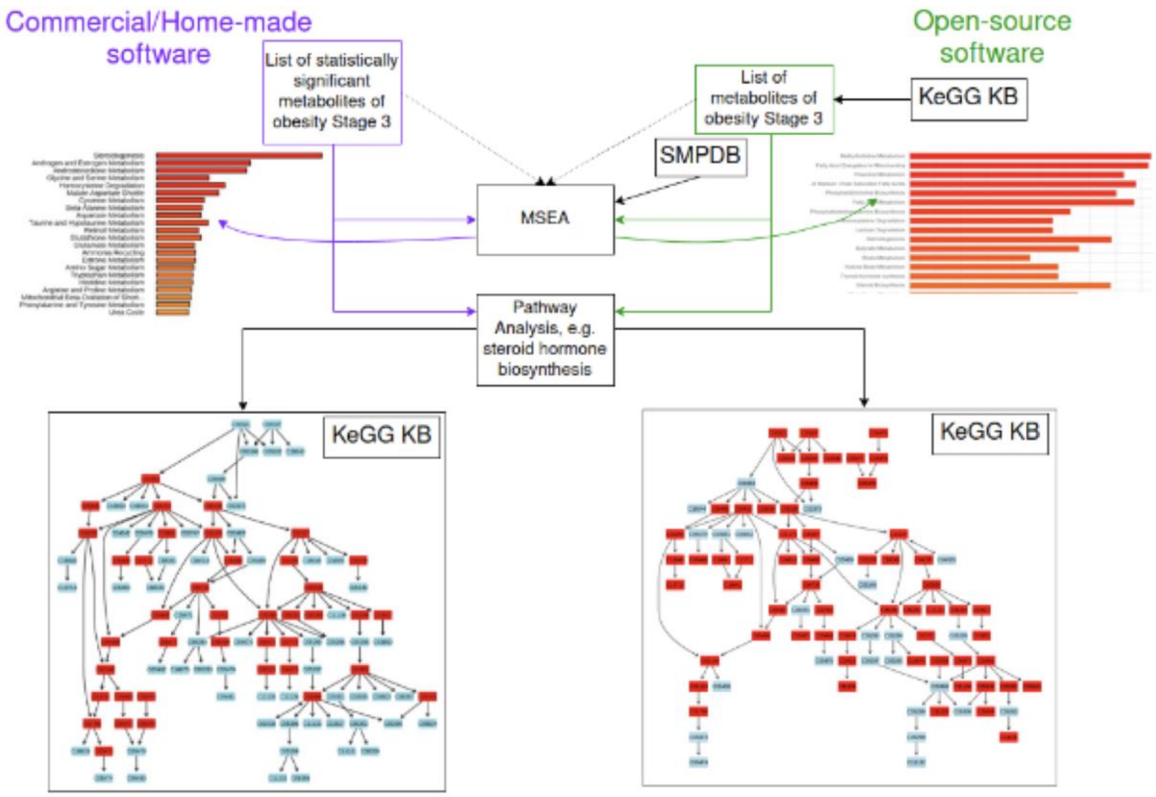

Figure S1. The scheme of algorithms for processing mass-spectrometric data of direct infusion. Utilizing the commercial program COMPASS DataAnalysis in junction with MatLab interactive development environment is shown on the left (purple). On the right (green) — open-source R packages: MSnbase, MALDIquant, MetaboAnalystR::Mummichog.
